# Adapting a skills-based stroke prevention intervention for communities in Ghana: a qualitative study

**DOI:** 10.1186/s43058-020-00084-8

**Published:** 2020-11-17

**Authors:** Temitope Ojo, Nessa Ryan, Joel Birkemeier, Noa Appleton, Isaac Ampomah, Franklin Glozah, Philip Baba Adongo, Richard Adanu, Bernadette Boden-Albala

**Affiliations:** 1 Department of Social and Behavioral Sciences, New York University School of Global Public Health, New York, NY USA; 2 Program in Public Health, Susan and Henry Samueli College of Health Sciences, University of California, Irvine, CA USA; 3 Global Health Program, New York University School of Global Public Health, New York, NY USA; 4 Department of Population Health, New York University Langone Health, New York, NY USA; 5 Concern Health Education Project, La-Accra, Ghana; 6 Department of Social and Behavioral Sciences, University of Ghana School of Public Health, Accra, Ghana; 7 Department of Population, Family and Reproductive Health, University of Ghana School of Public Health, Accra, Ghana; 8 Departments of Health Society and Behavior and Epidemiology, Program in Public Health, Susan and Henry Samueli College of Health Sciences, University of California, Irvine, CA USA; 9 Department of Neurology, School of Medicine, University of California, Irvine, CA USA

**Keywords:** Hypertension, Stroke, Pre-implementation, Intervention adaptation, Cultural adaptation, Proctor’s taxonomy of implementation outcomes, Qualitative methods, Ghana

## Abstract

**Background:**

Stroke is a major cause of death in Ghana. Evidence-based interventions for stroke prevention have been successful in the US; however, in low- and middle-income countries (LMICs), such interventions are scarce. The “Discharge Education Strategies for Reduction of Vascular Events” (DESERVE) intervention led to a 10-mmHg reduction in systolic blood pressure (SBP) among Hispanic survivors of mild/moderate stroke and transient ischemic attack (TIA) at 1-year follow-up. Our objectives were to capture the perceptions of a diverse set of stakeholders in an urban community in Ghana regarding (1) challenges to optimal hypertension management and (2) facilitators and barriers to implementation of an evidence-based, skills-based educational tool for hypertension management in this context.

**Methods:**

This exploratory study used purposive sampling to enroll diverse stakeholders in Accra (*N* = 38). To identify facilitators and barriers, we conducted three focus group discussions: one each with clinical nurses (*n* = 5), community health nurses (*n* = 20), and hypertensive adults (*n* = 10). To further examine structural barriers, we conducted three key informant interviews with medical leadership. All interviews were audio recorded and transcribed. Thematic analysis was carried out via deductive coding based on Proctor’s implementation outcomes taxonomy, which conceptualizes constructs that shape implementation, such as acceptability, adoption, appropriateness, cost, and feasibility.

**Results:**

Findings highlight facilitators, such as a perceived fit (appropriateness) of the core intervention components across stakeholders. The transferable components of DESERVE include: (1) a focus on risk knowledge, medication adherence, and patient–physician communication, (2) facilitation by lay workers, (3) use of patient testimonials, (4) use of a spirituality framework, and (5) application of a community-based approach. We report potential barriers that suggest adaptations to increase appropriateness and feasibility. These include addressing spiritual etiology of disease, allaying mistrust of biomedical intervention, and tailoring for gender norms. Acceptability may be a challenge among individuals with hypertension, who perceive relative advantage of alternative therapies like herbalism. Key informant interviews highlight structural barriers (high opportunity costs) among physicians, who perceive they have neither time nor capacity to educate patients.

**Conclusions:**

Findings further support the need for theory-driven, evidence-based interventions among hypertensive adults in urban, multiethnic Ghana. Findings will inform implementation strategies and future research.

Contributions to the literature

- Provides a unique pre-implementation narrative on using educational interventions to improve health outcomes for people who have had a stroke/TIA (secondary prevention) and those at risk of having a stroke due to hypertension (primary prevention) in LMIC settings
- Provides a model for a community-based approach to cultural adaptation of a stroke prevention program
- Applies Proctor et al.’s implementation outcomes in the pre-implementation period of an intervention
- Highlights the distinct relevance of early stakeholder engagement in the adaptation and the pre-implementation stages of an intervention

## Background

Stroke is a major global health concern. The rapid upsurge of stroke and other non-communicable diseases (NCDs) over the last few decades, especially in low- and middle-income countries (LMICs), is a significant public health challenge given the double burden of serious chronic illnesses and infectious diseases, such as malaria, tuberculosis, and HIV. Approximately 85.5% of all stroke deaths occur in LMICs [1]. In sub-Saharan Africa, stroke is characterized by young age of onset and a high propensity of being hemorrhagic, as well as high mortality and post-stroke complications [2]. Ghana is one example of an LMIC that is currently experiencing an epidemiologic transition, with an accompanying rise in its burden of stroke. Stroke is the second leading cause of noncommunicable disease (NCD) death in Ghana, with a 7.5% mortality rate [3] and an average stroke incidence age of 63.7 years [4]. Hospital admissions due to stroke have increased substantially in the past 30 years, from an admission rate of 5.3/1000 persons in 1983 to 13.9/1000 persons in 2013, and a mortality secondary to stroke increment from 3.4/1000 persons to 6.6/1000 persons with an average 28-day mortality of 41.1% [5].

Hypertension, a primary risk factor for stroke, is also highly prevalent in Ghana, estimated at 36.4% among adults aged 25+ [6]. Hypertension incidence is also rising rapidly, with a documented tenfold increase in diagnosed cases in Ghana’s outpatient public health facilities between 1988 and 2007 [7]. Of note, most hypertension cases in Ghana are undiagnosed as many Ghanaians do not routinely see their primary care provider, and the vast majority of cases are uncontrolled [8]. A substantial proportion of disability in Ghana is attributed to stroke, given that 50.0% of survivors are chronically disabled [9–12]. Most health facilities in LMICs such as Ghana have limited diagnostic capacity and limited resources, which further complicates the management of stroke [4]. According to Ghana’s Ministry of Health’s Standard Treatment Guidelines of 2010, most Ghanaian adult patients do not experience symptoms indicative of hypertension and are diagnosed by chance during a medical visit and examination. This phenomenon would explain the significant proportion of adults who are unaware of their hypertensive status during community-based and population-level prevalence studies conducted by independent researchers [13]. When diagnosed, most Ghanaian hypertensive patients would need two or more medications to achieve the desired blood pressure (BP) levels, which are below 140/90 mmHg for non-diabetic individuals or below 130/80 mmHg for diabetic individuals. Medical practitioners also recommend non-pharmacological treatment for hypertension, which addresses diet and lifestyle changes. Patients unable to achieve BP control or desired BP levels (with or without medications); those with one or multiple comorbidities such as diabetes, obesity, dyslipidemia, and with a family history of hypertension; or pregnant women are referred to a specialist to address treatment challenges [14]. In theory, the National Health Insurance Scheme in Ghana should cover or reduce costs for some level of hypertension treatment, including medications, but the reality differs as about 30% of Ghanaians are not covered under the insurance scheme [15].

Downstream prevention strategies focused on vascular risk reduction are almost nonexistent. Given the remarkable rise in NCD and stroke-related deaths in Ghana, it is clear that major efforts in primary and secondary prevention are needed. Although the Ghanaian public has been aware of the rise in NCDs since the 1990s, there are limited policies or initiatives in place to address this growing epidemic [16]. In the epidemiological transition from a sole burden of communicable diseases to an increasing burden of NCDs in LMICs, stakeholders must rely not only on structural changes but also on population-level behavior change. In the face of challenges such as lack of infrastructure and of financial instability, this dichotomy is particularly important, although complex and challenging.

Evidence-based educational and behavioral interventions, effective in high resource settings and among certain ethnic groups when culturally adapted, have not been introduced in this context. Strategies to address this complex challenge require community and other stakeholder engagement, which informs relevant tailoring for a population, as well as infrastructure to affect population-level behavior change. Using approaches grounded in implementation science, these effective interventions could be translated to the Ghanaian context to produce significant reductions in the stroke burden. One such intervention is the Integrated Care for Reduction of Secondary Stroke (ICARUSS) model implemented in Melbourne, Australia among survivors of transient ischemic attack (TIA) or completed stroke [17]. This randomized controlled trial evaluated the efficacy of an intervention which integrated patient education from a clinical coordinator and bidirectional flow of clinically important information between stroke specialists and primary care physicians to promote vascular risk factor management on risk factor modification, lifestyle changes, patient education, and disability of stroke survivors [17]. Results showed that the intervention decreased systolic blood pressure (SBP) significantly at 12 months’ follow-up, with a clinically and statistically significant 6.0 mmHg SBP reduction in the integrated (treatment) care group, compared to the 1.8 mmHg SBP increment in the control group that received standard care. Race/ethnicity was not reported and only English-speaking patients were targeted. Another intervention with a greater health equity focus, the recently completed Discharge Education Strategies for Reduction of Vascular Events (DESERVE) study, was implemented in New York, US in a multi-ethnic cohort of mild/moderate stroke and TIA survivors [18]. This randomized controlled trial assessed the efficacy of a culturally tailored, skills-based educational intervention on secondary stroke prevention. The DESERVE intervention, based on the Trans Theoretical Model of behavior change that presents behavior as dynamic and prioritizes self-efficacy, was composed of motivational videos, a skills-based workbook, an interactive educational session led by a community health worker, and follow-up communication (see Table 1) [19]. The DESERVE intervention addressed three themes: patient-physician communication, medication adherence and accurate risk perception [19].

**Table 1:** Intervention summary and mode of delivery

	Baseline	Discharge	72 h	1 month	3 months	6 months	12 months
Intervention group	• Baseline interview	• Educational PowerPoint Presentation • Motivational Video	• Post-stroke contact form • Delivery of workbook • Verify follow-up doctor’s visit	• One-month follow-up form • Reminder about doctor’s visit • Review patient/physician communication in workbook • Follow-up call after doctor’s visit	• Three-month follow-up form • Follow-up other doctor’s visits • Review main component of workbook • Discuss other resources	• In-person visit/form • BP, HbA1c, Anthropometrics • Re-review workbook	• In-person visit/form • BP, HbA1c, Anthropometrics • Re-review workbook
Standard care group	• Baseline interview	• Standard discharge education	• Patient-guided contact	• Patient-guided contact	• Patient-guided contact	• In-person visit/form • BP, HbA1c, Anthropometrics	• In-person visit/form • BP, HbA1c, Anthropometrics

This table shows the intervention summary and mode of delivery during the trial in the USA

*BP* blood pressure, *HbA1c* glycated hemoglobin

A prominent feature of cultural adaptation of DESERVE was the use of a spirituality framework, where the Spanish versions of motivational videos were tailored to the notions of faith and community. Results showed a major impact of the intervention on SBP reduction at one year follow-up, with a clinically and statistically significant 9.9 mmHg greater SBP reduction in intervention vs control group among Hispanic participants [18]. The DESERVE study highlights the potency of culturally tailored, skills-based education in achieving sustained risk factor control. There are several factors of the Hispanic participants’ stroke recovery experiences that are reminiscent of the typical experience of Ghanaian individuals living with hypertension, which puts them at high risk for stroke. Like the Hispanic population in the US, Ghanaians living with hypertension have limited stroke-specific health literacy and access to stroke risk reduction services and resources [4, 8]. In addition, a large proportion of the Ghanaian society affiliates with faith-based organizations and attends faith-based activities [20, 21]. A prior study has also shown that among Ghanaians, groups that put more importance on religion had a more pleasant experience of subjective well-being [22]. The Hispanic DESERVE intervention materials were culturally adapted, with the help of community members, to frame stroke recovery in the context of faith and spirituality. This, among other factors addressing access to stroke resources, could be responsible for the adherence to the DESERVE intervention and statistically significant reduction in SBP among the Hispanic participants, which was not observed in the non-Hispanic African American and White participants [18]. Additionally, we believe that as the comparison group for the RCT was enhanced care rather than standard of care, a larger study would be needed to observe the likely positive impact across all participants but that the signal was strongest among Hispanic participants. We believe this was because materials had been linguistically and culturally adapted for that group and using an equity lens, Hispanic participants likely had greater challenges to overcome, in terms of access, risk, communication, and medication adherence. We therefore hypothesized that culturally adapting the DESERVE intervention for adults with hypertension in Ghana will lead to significant vascular risk reduction for this high-risk group.

The analytic framework for this work was developed from Proctor et al.’s taxonomy of outcomes for implementation research (see Table 2) [23]. Given the exploratory nature of this study towards the adaptation of the DESERVE intervention for the Ghanaian setting, Proctor’s implementation outcomes are desired outcomes we want to examine early in the planning and preparation processes of intervention adaptation and implementation. By characterization, Proctor’s taxonomy of implementation outcomes are proximal indicators of implementation process, and as such, it is fitting to identify the presence or absence of these outcomes, as well as factors that would enable or deter their desired presence as we adapt DESERVE and commence implementation. We focus on identifying traits of outcomes, namely acceptability, appropriateness, adoption, and feasibility that are salient to the early stage of the implementation process and likely to improve the potential for implementation success, if attained through the process of adapting and implementing the intervention. If interventions are to be successfully adapted for a novel cultural context, as evidenced through observation of desired changes in clinical or service outcomes, these interventions need to be implemented well through purposive actions that would reflect through stakeholders and participants accounts that the intervention is acceptable, appropriate, feasible, and can be adopted in the target setting. This exploratory study aimed to capture the perceptions of a diverse set of stakeholders in an urban community in Ghana regarding challenges to optimal hypertension management and facilitators and barriers to implementation of an evidence-based, skills-based educational tool for hypertension management in this context.

**Table 2:** Definitions of implementation outcomes

Outcome	Definition
**Acceptability**	**The perception among implementation stakeholders (beneficiaries and implementers) that the innovation is agreeable, palatable, or satisfactory**
**Adoption**	**(Uptake) The intention, initial decision, or action (here we are interested in the behavioral intention) to try or employ the innovation**
**Appropriateness**	**The perceived fit, relevance, or compatibility of the innovation for a given practice setting, provider, or beneficiary; and/or perceived fit of the innovation to address a particular issue or problem (here we are interested in hypertension)**
**Costs**	**(Incremental or implementation cost) The cost impact of an implementation effort**
**Feasibility**	**The extent to which the innovation can be successfully used or carried out within a given agency or setting**
Fidelity	The degree to which the innovation can be implemented as it was prescribed in the original protocol or as it was intended by the program developer
Penetration	The integration of a practice within a service setting and its subsystems
Sustainability	The extent to which a newly implemented innovation is maintained or institutionalized within a service setting’s ongoing, stable operations

Based on Proctor et al.’s framework for implementation outcomes (text in bold emerged within our analysis)

## Methods

### Study design

This multi-method qualitative study with diverse stakeholders in Accra (*N* = 38) utilized three focus group discussions, with clinical nurses (*n* = 5), community health nurses (*n* = 20) and hypertensive adults (*n* = 10), to assess facilitators and barriers to an educational intervention to address hypertension in this context. To further examine structural barriers, we conducted three key informant interviews with medical leadership. The Consolidated criteria for Reporting Qualitative Research (COREQ) checklist [24] was used to report this study, given the exclusive use of qualitative methods to conduct the exploratory aspects.

### Description of study setting

The La Dade-Kotopon (La) Municipal District is one of 26 administrative districts under the Greater Accra Regional Health Directorate in Ghana. La Dade-Kotopon Municipal Assembly is the local authority, where medical and administrative leadership oversee the health affairs and services for the district. La has a general hospital, La General Hospital, which serves as a model institution for the Ghana National Health Insurance Scheme (NHIS) and provides health services to the most populated region in the country. Korle Bu Teaching Hospital (KBTH), located in Ga East District, is the premier and largest tertiary care referral center in Ghana, the third largest hospital in sub-Saharan Africa.

### Participant recruitment

Purposive sampling was used to recruit and enroll diverse stakeholders (*n* = 38), comprised of medical leadership from La Municipal Assembly, La General Hospital, and KBTH; healthcare providers from these institutions; and adults living with hypertension within the La community in Accra. Community nurses and clinical nurses were recruited from the Health Directorate of La Municipal Assembly and the La General Hospital, respectively. Adults living with hypertension were recruited from a previous community BP screening project conducted by the research team in 2017, in partnership with a local non-governmental organization (NGO), Concern Health Education Project. The community screening program checked BP using Omron blood pressure machines which are Federal Drug Administration-approved for clinical accuracy. Uncontrolled hypertension was defined as having an average SBP of 140 mmHg or higher or an average diastolic blood pressure (DBP) of 90 mmHg or higher [25]. Controlled hypertension was defined as achieving an average SBP less than 140 mmHg or DBP less than 90 mmHg, in individuals who have been previously diagnosed with hypertension. Previous participants were sent invitation letters to participate in this study. Eligible individuals were members of the La community with self-reported controlled or uncontrolled hypertension, between the ages of 18 to 80 years old. Our partner NGO, Concern Health, assisted with the recruitment of participants for all focus group discussions. Four research team members (TO, NA, JB, and IA) consented eligible participants face-to-face, first by explaining the purpose of the study, study expectations of participants, study benefits, minimal risk associated to the study, and voluntary participation in the study. After this, participants signed consent forms and were given a copy of the consent to keep.

### Data collection and analysis

To identify facilitators and barriers to implementation of the DESERVE intervention, we conducted three focus group discussions: one each with clinical nurses (*n* = 5), community health nurses (*n* = 20), and hypertensive individuals (*n* = 10). Questions within the discussion guide were adapted based on the type of participant being addressed, but the general structure and order of the domains were the same. The domains included identifying factors that contribute to inadequate hypertension management in this context, suggestions for an educational intervention to address hypertension in Ghana, and perceptions of key DESERVE intervention components, such as using motivational storytelling of successful hypertension management, delivering the intervention in audio or visual format, and employing a points-based system to motivate and measure progress in meeting individual goals. Prior to asking about the DESERVE intervention specifically, the moderator presented a brief summary of that intervention and how it had been delivered previously in the US. Some sample questions included:

For Hypertensive individuals

- How do people in your community believe that hypertension can be treated or prevented?
- When thinking about hypertension prevention, in what setting are people most likely to listen to health advice?
- What type of program would help motivate you to engage in hypertension prevention practices on a regular basis?
- How do you feel about the use of point systems and grades to help motivate you for changing health behavior?
- Do you talk to your friends and/or family members about health and if so, what do you discuss?

To gain expert insight into the existing health system and to further examine structural barriers to hypertension management, we conducted three key informant interviews with a representative of medical leadership, a respresentative of nursing leadership, and a cardiologist. The structure and domains within the interview guides were similar to the focus group discussion script; however, the interviewers additionally reported emerging themes from the discussions to the key informant interviewee to seek their input on interpretation or clarification. All focus group discussions and key informant interviews were conducted by at least three research team members. One team member (TO) moderated all the sessions for consistency while the other team members (NA, JB, IA, and FG) took notes and probed participants to clarify and expand on certain questions, which produced detailed responses from participants. All sessions were audio-recorded. On average, key informant interviews lasted for 37 min (range 27–44) and focus group discussions, 67 min (range 43–74). A bilingual interpreter in English and Ga, working with the partner local NGO, assisted in moderating the focus group discussions with adults with hypertension, as participants were more fluent in Ga than in English.

Two research team members (TO and NR) transcribed the audio recordings and provided a narrative summary of each session. Narrative summaries were created after each interview was transcribed to help the research members document their thoughts and to highlight key sensitizing concepts that emerged from the interviews during the transcription process. Because the sample contained different types of stakeholders, the summaries helped to encourage comparison across stakeholder type. Transcripts were then thematically analyzed in NVivo v.12 via deductive coding based on Proctor et al.’s outcomes for implementation research [23], which conceptualizes the constructs of acceptability, adoption, appropriateness, cost, feasibility, fidelity, penetration, and sustainability. The last three implementation outcomes did not emerge when coding as they are less relevant for the pre-adoption period. Deductive coding was followed by inductive coding of themes not already identified based on the conceptual framework, including a theme related to multilevel factors contributing to suboptimal hypertension management and a theme related to concepts with the updated Framework for Reporting Adaptations and Modifications-Enhanced (FRAME) [26]. Techniques to enhance trustworthiness included debriefing with experts and developing an audit trail.

The study was approved by New York University Institutional Review Board (IRB-FY2018-1420), Noguchi Memorial Institute for Medical Research Institutional Review Board (IRB 00001276), and Ghana Health Services Research Ethics Review Committee (GHS-ERC 016/05/19).

## Results

Our findings present the challenges for optimal hypertension management among an urban community in Ghana, as well as the factors that might challenge or facilitate implementation of an adapted DESERVE intervention in this context. We compare and contrast across stakeholders. The results are presented hierarchically by Proctor et al.’s implementation outcomes that most prominently emerged among stakeholders, as evidenced by the frequency and depth with which those outcomes were discussed, including acceptability, appropriateness, adoption, cost, and feasibility [23].

### Sample characteristics

The sample characteristics are identified in Table 3. In the focus group discussion with community members with hypertension, mean age was 54.6 years (range 28–77) and the group consisted of two men and eight women. Of the 20 community nurses who participated in one of the health provider discussions, only two were male. All the clinical nurses in the second health provider discussion were female. Two of the three medical leaders who were interviewed were male.

**Table 3:** Sample characteristics for participants in focus group discussions and stakeholder interviews

Focus group discussions		
Clinical nurses (*n* = 5)	All females	Clinical nurses at La-General Hospital within La Dade-Kotopon
Community health nurses (*n* = 20)	2 males, 18 females	Nurses integrated to help support the health system within the community to promote preventive health measures
Hypertensive individuals (*n* = 10)	Mean age: 54.6 years (range 28–77 years); 2 males, 8 females	Community members in La Dade-Kotopon with hypertension that was controlled or uncontrolled, with some reporting current use of antihypertensives
**Stakeholder interviews**		
Participant 1: nursing leadership	Female	Leader at La Kotopon District Assembly
Participant 2: cardiologist	Male	Trained cardiologist at KBTH
Participant 3: clinic leadership	Male	Leader at La General Hospital within La Dade-Kotopon

### The need for a hypertension intervention

**Fig. 1:**
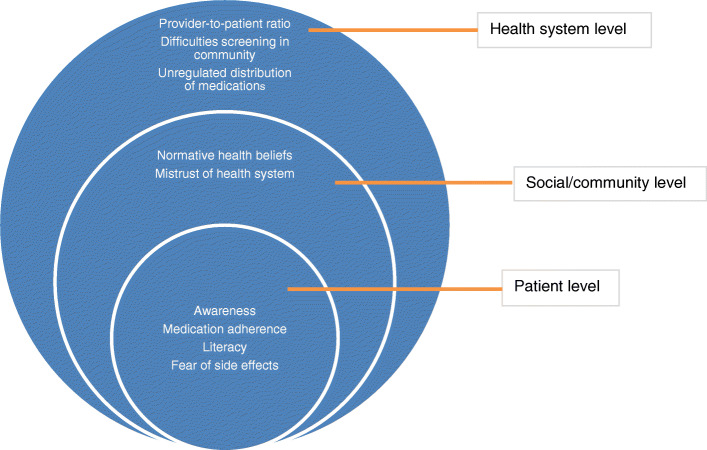
Multi-level factors contributing to poor uptake and maintenance of interventions for hypertension management in the Ghanaian context. This figure charts the multi-level contributing factors for poor uptake and maintenance of interventions for hypertension management in the Ghanaian context. Participants identified these factors within focus group discussions and key informant interviews and researchers mapped these onto a socio-ecological framing during analysis. This highlights that these factors play out at the level of the patient, social, community, and health system

All stakeholders could identify the need for a primary and/or secondary stroke prevention effort in their community. Tertiary prevention, as one clinician described, was not prioritized in this context, as early hospital admission and mechanistic clot removal are not feasible. Discussions began by identifying the factors that contribute to inadequate hypertension management in this context (see Fig. 1), which ranged from the level of the individual to the health system.

Various individuals with hypertension identified genetics, poor diet, alcohol consumption, smoking, stress, pregnancy, and aging as causative factors for hypertension. Clinicians felt that patients are aware they have been diagnosed with hypertension and need to take medicine, but the patients do not know about the contribution of adopting healthier lifestyle habits or how to check their BP. Nurses are already tasked with educating patients, although challenges exist for follow through (i.e., limited time to spend with patients in the clinical facility) and follow-up (i.e., community health nurses may have difficulties accessing hypertensive individuals or lack BP screening equipment). Primary prevention efforts included education on risk reduction through diet and physical activity during community outreach. Ongoing secondary prevention efforts included multi-screening for hypertension and comorbidities (i.e., diabetes) during routine visits to the hospital’s accident and emergency department and singular BP screening administered by community nurses or physician assistants at Community-Based Health Planning and Services or CHPS, which are community-based health centers in Ghana. Additionally, clinicians expressed frustration with pharmacies that provide incorrect dosage and traveling salesmen in the community who offer BP screening and what they refer to as BP medications. These are services that are unregulated and presumed of low quality by clinicians. Although antihypertensive medications are available, including calcium channel blockers and diuretics, stakeholders reported significant challenges for individuals to initiate and maintain medications, due in part to low levels of patient-provider communication, patients not being able to afford medications, and insufficient utilization of the NHIS for coverage of some medication costs.

### Facilitators and barriers to implementation of an adapted DESERVE intervention

In conceiving of implementation of a skills-based educational intervention to prevent stroke, stakeholders identified a variety of potential facilitators and barriers across the five key implementation outcomes: (1) acceptability, (2) appropriateness, (2) adoption, (4) cost, and (5) feasibility. Facilitators and barriers are described below and presented in Table 4.

**Table 4:** Facilitators and barriers to implementation of a culturally adapted skills-based stroke education tool

	Facilitators	Barriers
**Implementation outcomes**		
**Acceptability**	**Attitudes/burden/self-efficacy/intervention coherence**	
	**Attitudes** • Participation motivated by desire to care for self and children/grandchildren^a^ **Burden** • Delivered as one-on-one discussion from provider, potentially with patient’s partner or caretaker^c^ • Community nurses already going out in communities to address people’s fears of the medical system^e^ **Self-efficacy** • Some hypertensive community members already seeking information/advice from multiple sources^a,b^ **Intervention coherence** • Successful hypertension management modeled to patients through images and stories^b,c^ • Show how people struggle with stroke^c^ • Provide audio aids for patients to take home with them^e^	**Attitudes** • Religious beliefs encourage “*preemptive rejection*”/denial of ill-health^c,f^ • Spiritual etiology of ill health is normalized and requires spiritual intervention^a,b,c,d,e,f^ • Some fear hypertension diagnosis caused by BP screening^a^ • Experienced or anticipated medication side effects for men (erectile dysfunction, low libido, “feeling uncomfortable”)^a,c,d,e^; for women, diabetes onset^a^ **Burden** • Patients screened in the community expect to be treated on the spot, rather than receiving a referral to the hospital^e^ **Self-efficacy** • Self-efficacy regarding taking local herbs/alternative medicines, not hypertensive medications^a^ **Intervention coherence** • Locus of control—some nurses see the patient’s lack of discipline as reason for their inability to adhere to medication regimen^b^—this was not mentioned by patients^a^
**Adoption**	**Implementers**	
	• Community and clinical nurses see themselves implementing, with training^b,c,e^	• Physicians do not perceive themselves as best implementers^d,f^
**Appropriateness**	**To address hypertension**	
	• Screening and education should be delivered together	
	**For potential beneficiaries**	
	• Use simple language and translate in different languages^c^ • Utilize a cultural insider/community member to deliver health message^d^ • Successful implementation is more likely if delivered by community opinion leaders,^c,d^ particularly the church^c^ • Culturally significant gender roles and expectations important for tailoring messaging^a,b^	
	**For implementers**	
	• Educators should warmly welcome patients at the beginning of the educational session^c^	• Physicians do not perceive they have time to educate^b,c,d,f^ • Community nurses have varying levels of training and ability to educate patients^d^ • Community nurses have broken equipment to monitor BP in the community^b,e^
	**For setting**	
	• Places in which the intervention could be implemented: at home, in the clinic, at market, at clan meetings, at durbars, in schools, and at church^b,c,d,e,f^	• Unregistered salesmen, who may even refer to themselves as “doctor,” going around in the community offering to check BP, then using the opportunity to sell drugs to individual who may or may not need it^b,c^ • The herbalist has time to educate, the clinician does not^d^ • Community health nurses need transportation to reach communities in need^b^ • Not enough BP machines in community setting^b,e^ • Nurses are already trying to screen and educate in the community, but lack educational materials (i.e., fliers, posters or visuals showing conditions due to hypertension), logistics and funds^e^ • Community distrust of the biomedical establishment^b,d^ • Relative advantage of alternative health systems (herbalism, use of natural foods like pepper or ginger, traditional medicine, Chinese medicine, prayer camps), which promise total cure and easier dosage while biomedical approach requires continuous management^b,c,e,f^ • Patients fear maltreatment in the medical system^b,c,e^ • Patient inconveniences like long wait times, language barriers^b^
**Cost**	**Opportunity cost**	
		• Physicians do not perceive they have time to educate patients^b,d,f^
	**Patient cost**	
		• In spite of knowing their hypertension status, some people cannot seek treatment due to poverty^e**,**f^ • Patients report cost of having to go monthly to renew prescription^a^ • Cost for medications not covered by NHIS^f^ • Although medications are supported by NHIS, many people do not register due to sense of fatalism^b^ or mistrust of the health system^c^
	**Implementation cost**	
		• Nurses lack funding for educational materials and maintaining BP screening equipment^e^
**Feasibility**	• Feasible to implement in clinical or community setting^d^ • Some presence of social support to manage hypertension among close family and friend networks^a^	• Community health workers have variable capacity for educating due to their training^d^ • Perceived lack of healthcare support by hypertensive individuals given poor provider-patient communication^a^ • Lack of a seamless continuum of care^a,b,e,f^

^a^Focus group discussion (FGD) hypertensive patients

^b^FGD community health nurses

^c^FGD clinical nurses

^d^Key informant interview (KII) medical leadership

^e^KII nursing leadership

^f^KII cardiologist

#### Acceptability of intervention

The *prospective acceptability* of this educational intervention assessed in the pre-implementation period was balanced with both facilitators and barriers. There were perceptions among stakeholders (i.e, clinicians, administrators, and potential beneficiaries) that the intervention was agreeable prior to participation in the intervention [23, 27], and stakeholders made suggestions of how hypothetical acceptability could be improved. Hypothetical acceptability is a multifaceted construct that touches on anticipated cognitive and emotional responses to the intervention, including *attitudes* (feelings about the intervention), *burden* (perceived amount of effort to participate), *self-efficacy* (individual confidence that they can perform the behavior required of the intervention), and *intervention coherence* (understanding of the intervention and its components) [27].

Hypertensive individuals and clinicians alike reported that potential beneficiaries would find this educational intervention acceptable in the Ghanaian context, as hypertensive individuals are already seeking out information on their hypertension and advice regarding its management (i.e., from peers, the Internet, and clinicians). As far as format of educational materials, community, and clinical nurses suggested animations and/or patient testimonials would be acceptable (as they were acceptable for nationally televised campaigns for cholera and malaria). Community health nurses reported “seeing is believing” as they thought patients would respond well to observing ambassadors with hypertension who are successfully managing the condition. One clinical nurse reports: “They need to see how people struggle with stroke. Just telling them is not enough.” Hypertensive individuals reported positive attitudes towards the intervention, as they were motivated by a desire to take care of themselves and their progeny, as well as some negative attitudes, mostly regarding fear of side effects from antihypertensive medications, especially among male hypertensive individuals who complained of erectile dysfunction (ED) as a deterring side effect. Threats to hypertensive individuals’ acceptability also included the perceived burden of not being able to offer treatment at the point of screening in the community setting and a comparatively higher self-efficacy towards herbal remedies than towards antihypertensive medications. Resoundingly, religious teachings and normative beliefs regarding spiritual etiology of disease were reported by all stakeholders as barriers to acceptability among potential beneficiaries.

#### Appropriateness of intervention

The intervention appropriateness, or the perceived fit of the intervention within the Ghanaian context, was assessed for the potential beneficiaries, implementers, and setting, as well as for its relevance to address the issue of hypertension. An educational intervention for hypertension management and stroke prevention is relevant in this context, as supported by the fact that nurses report current attempts to educate patients on risk factors, antihypertensive medications, and the chronicity of hypertension when they have time and resources. As illiteracy is high in some communities and Ghana has over 100 dialects, stakeholders report appropriateness for potential beneficiaries can be improved by using simple language (i.e., absent of jargon), visual aids, and translating educational materials into different languages. A health message delivered by a cultural insider or community member is more likely to be successfully received by beneficiaries. According to both hypertensive individuals and clinicians, the health message should also be tailored for gender roles and expectations in this context, as gendered differences in ideal body weight (i.e., overweight body size for men is a positive status symbol) and different risk factors related to pregnancy were reported.

Importantly, a potential pushback to implementation efforts is that physicians do not perceive they have the time to educate their patients well or sometimes even at all in busy clinical settings. Community health nurses may have varying levels of training/qualification and access to BP monitoring equipment; therefore, not all perceive they have the tools or training to screen and educate hypertensive individuals in the community. However, nurses did feel appropriateness could be improved by making patients feel welcome and relaxed at the beginning of the educational session, potentially educating community members in their homes where they are more receptive, as well as in clinical facilities where patients are attentive as they need help. Additional suggestions for community-based settings for implementation include markets, clan meetings (meeting of a tribal group), durbars (community meeting led by traditional leaders), schools, and churches. One representative of nursing leadership suggests:

“Nurses and doctors should make [the intervention] available at hospitals. Public health and community nurses will make it available within the communities. We go from house-to-house to visit the aged, people with communicable and NCD conditions, and provide education. There are some NGOs in the communities giving home visits and give health services. When they come across complicated cases, they refer people to clinics….We will have to educate [hypertensive patients] to know the ‘inside’ of their condition and the complications attached to it and how fatal it is. They don’t want to die. If you let the person know the outcome of non-adherence to antihypertensive medications as being fatal, they will comply…Upon diagnosis, a person should be educated on the situation, what they are supposed to do, how to go about treatment, how long to take treatment, and when to come for reviews. Community nurses will also follow up at homes to check on their wellbeing, how they are doing on the meds, side-effects, make referrals if needed, and making sure the patients go for their reviews. We also tell them not to wait until the last pill to come for a refill prescription.” (Key informant interview, nursing leadership)

#### Adoption of intervention

Adoption, defined here as the intention to employ the educational intervention, was assessed at the level of the provider and organization, and both facilitators and barriers to intended uptake were identified. There is a feeling that the situation is untenable, in that patient education is needed but there is little capacity in the current health system (for physicians) to provide education. Community health nurses reported the patient-provider relationship is already strained due to the low provider-to-patient ratio, patient frustration from long wait times, language barriers, and chastising of patients by nurses for poor BP readings. Community and clinical nurses reported that they could, with appropriate training, implement the intervention. Although physicians may not implement the educational intervention, they would support evidence-based intervention, as demonstrated by one physician:

“Educate individuals with various diseases, go on TV, market places, church, buses, NGOs… individuals, hospitals, government agencies... We should be reaching [community members] at the local level by using people within the community to go round, pick and train them from the community. Those who are unemployed but are trained nurses or university graduates can be educated to educate the general community. It requires massive investment to be sustainable... The community will listen to them more and be able to link people up to the formal health structure and the NHIS. This will be more effective than broad stroke advertising and education campaigns…These people will go from door-to-door with BP machines, check their BP, and identify people with hypertension or diabetes and link them to health workers. It worked in Chennai, India, where they did massive health screening for NCDs and it reduced incidence of NCDs.” (Key informant interview, cardiologist)

#### Cost of intervention

Cost was exclusively referred to as a barrier to implementation, most notably as an opportunity cost for physicians, who do not perceive they had the time to educate patients in their busy clinical facilities. Additionally, various patient costs were identified, including the cost of seeking treatment at a clinical facility, particularly the cost of having to renew their antihypertensive medication prescriptions every month. Although medications are supported by insurance provided by the NHIS, clinicians reported many people do not register for insurance due to a sense of fatalism or mistrust of the health system. Most people are covered by the NHIS for diuretics and calcium channel blockers, but people with comorbidities must pay out-of-pocket for some expensive medications that are not covered. Lastly, nurses reported a lack of funding for educational materials, including visual aids and fliers.

#### Feasibility of intervention

The extent to which DESERVE could be successfully used or carried out within a given setting was discussed within the context of whether it would be better to implement in the clinical or community-based setting. Stakeholders found benefits to both:

Patients are compliant to clinical attendance. Patients are likely to listen to the clinician in the clinical setting when they feel vulnerable, but less likely to listen to the clinician when they are well and at home. (Key informant interview, medical leadership)

Additionally, some patients reported receiving social support from network members (family and friends) to help them manage their hypertension (i.e., a friend helps one patient check her BP). Some barriers to feasibility were also reported, including the variable capacity among community health workers (CHWs) to educate patients and a perceived lack of healthcare support by hypertensive individuals given poor provider-patient communication. Feasibility could be threatened by disruptions in the continuum of care due to shortage of working BP machines for community screening, inconvenient protocol for medication prescriptions and refills, and gaps in insurance coverage for medication costs.

## Discussion

**Fig. 2:**
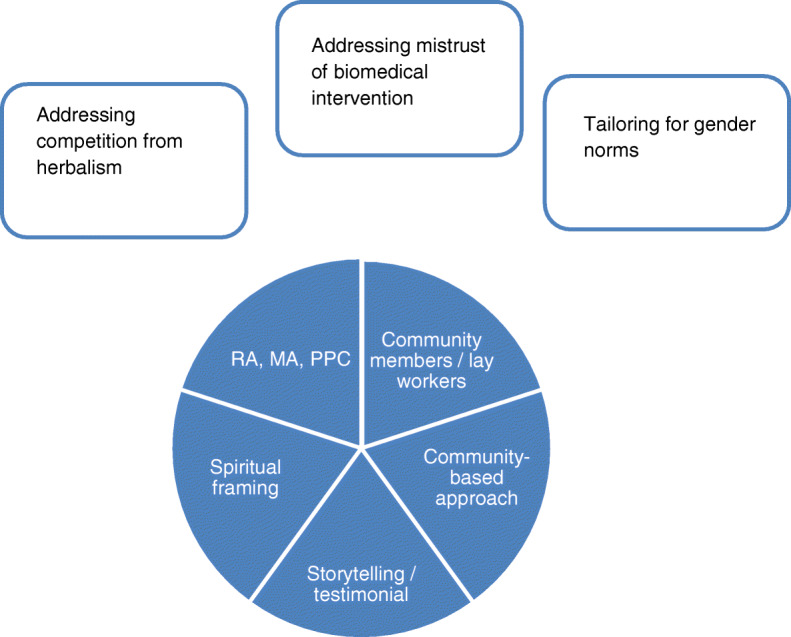
Core and tangential components of skills-based educational intervention for hypertension management in the Ghanaian context. This figure presents the concepts related to adaptation of the DESERVE intervention for the Ghanaian context. The core constructs, those which we believe must be maintained to ensure effectiveness, are indicated as the five quadrants within the blue circle. The tangential and modifiable components, those which we believe must be adapted to this new context, are presented in the boxes surrounding this circle. RA, MA, PPC = risk assessment, medication adherence, patient-provider communication

Findings highlight contextual factors and facilitators and barriers to implementation of a skills-based educational intervention to prevent stroke, which will further inform cultural adaptation of the DESERVE intervention to the Ghanaian context. Our findings suggest a perceived fit (appropriateness) across stakeholders of the core components of the intervention. In cross-cultural implementation of prevention efforts, successful translation is dependent on resolving the tension between the compatibility of the intervention within the new context and identifying core components to maintain in order to observe impact (if in fact the intervention is effective). Based on our formative assessment, the core components of the intervention (which will remain the same to maintain effectiveness) and the tangential components (which should be adapted for this context) are identified in Fig. 2.

The transferable components, which we refer to as the core components, of DESERVE include (1) focus on accurate risk assessment, medication adherence, and patient–provider communication, (2) facilitation by CHWs or lay workers, (3) utilization of patient testimonials, (4) use of a spirituality framework, and (5) application of a community-based participatory approach. We report potential barriers that suggest adaptions to increase acceptability, appropriateness, and feasibility. These include addressing spiritual etiology of disease, allaying mistrust of biomedical intervention, and tailoring for gender norms, as it relates to the recurring report of the male-specific side effect of erectile dysfunction. Acceptability may be a challenge among patients, who perceive relative advantage of alternative therapies like herbalism. This has been found with other biomedical innovations implemented in Ghana [28, 29] and throughout Africa [30]. Key informant interviews highlight structural barriers (high opportunity costs) among physicians, who perceive they have neither time nor capacity to educate patients. Nonetheless, key informant interviews support other evidence-based programs, such as a task-shifted, large-scale NCD screening program in Chennai, India, to increase linkage to care using onsite teleconferencing at the point of screening [31]. Addressing risk knowledge, medication adherence, and patient-provider communication [8, 32] with intervention delivery task-shifted to a lower cadre of workers than physicians within a community-based approach is an ongoing focus of hypertension interventions in Ghana [33, 34]; however, there has been limited focus on utilization of a social cognitive approach that prioritizes patient testimonials and a spirituality framework to deliver skills-based education focused on self-efficacy. Research supports the benefits of prioritizing patient perceptions in culturally tailored health promotion interventions, which focus on improving communication, information access, and stimulating lifestyle behavioral changes through skills-based education [35–37]. Research also supports the potential impact of a faith-based approach to hypertension management in black American populations [38, 39].

By focusing on gathering the input of a diverse set of stakeholders from the clinical and community setting to inform adaptation, we provide a model for a community-based approach to cultural adaptation of a stroke prevention program in an LMIC setting. Following the guidance of the FRAME framework for reporting adaptations to evidence-based interventions [26], we utilize a multi-method qualitative methodology to gather input from a diverse set of stakeholders during the pre-implementation period. The main reason for adaptation was to increase appropriateness for an LMIC setting. The US-based researchers, who were involved in the original DESERVE intervention, proposed modification and met with Ghanaian collaborators to proactively plan an adaptation, which was decided upon collectively once formative data was collected. Although the original DESERVE intervention was developed and trialed in a high-income country with a predominantly black and Hispanic population and is focused on secondary prevention for patients who have already had a stroke/TIA, the adapted DESERVE will focus on primary prevention as this upstream approach is more appropriate for a LMIC setting. The team is planning modifications that will be piloted in subsequent research, adaptations which include addressing competition from herbalism, tailoring for gender norms, and addressing biomedical mistrust. This research will need to examine fidelity to the adapted intervention. The educational intervention is delivered at an individual level, but will likely need to address health systems-level challenges in future iterations.

There are various strengths to this research, including its focus on implementation in the pre-adoption period, the community-based approach, and the early engagement of a diverse set of stakeholders (i.e., hypertensive individuals, health providers, and medical leadership). For instance, our local NGO partner displayed the capacity to complement the efforts of community health nurses to promote hypertension prevention and management within the communities, through its mobilization of diverse stakeholders for the study. There are also some limitations. The major limitation concerns the applicability of focusing on DESERVE and similar individual level educational interventions in health systems that are not patient-centered, or where the concept of individuality within a health care team is not compatible with the organizational culture. This work focused on adaptation over fidelity, and so cannot comment on stakeholder perceptions of fidelity to the DESERVE intervention. Future research, in an effort to contribute to implementation and prevention science, should examine the tension between adaptation and fidelity. The use of Proctor et al.'s taxonomy of implementation outcomes, though relevant as an exploratory framework for developing an implementation research study, does not offer an exhaustive platform to assess context-specific domains of adapting DESERVE for the Ghanaian population. Other implementation science frameworks, such as the Consolidated Framework for Implementation Research (CFIR), could have provided a more extensive and explanatory framework for evaluating the presence or absence of context-related determinants of implementation across the individual and health system levels [40].

## Conclusion

The DESERVE intervention’s theory of change posits that a strong provider-patient relationship (encouraged through communication) and better risk awareness (imparted by improved knowledge and self-efficacy) could lead to better adherence to medication and lifestyle change. In this context, we see that there may be some additional challenges, including mistrust of biomedical interventions, insufficient time and capacity for provider-patient engagement, subscription to spiritual etiology of hypertension and stroke, fear of reprimand from providers, and the lack of funds to refill medications. Therefore, it will be critical to create health messages that build trust in biomedical intervention, conduct sensitivity training on motivational communication between providers and patients, and train allied health workers to educate and counsel hypertensive patients. Though addressing the lack of funding for medications is not within the purview of this intervention, the early engagement of multiple stakeholders at the adaptation and pre-adoption stages of this intervention is an opportunity to build lasting buy-in from medical and administrative leadership. Suggested implementation strategies include adopting a community-based approach that will engage highly motivated individuals as community champions to model hypertension management and visibly engage community (cultural and spiritual) leadership to be involved in promoting values of the proposed intervention.

## Data Availability

Datasets on this study are available in the form of transcripts, which will be made available on reasonable request.

## Abbreviations

BBA: Bernadette Boden-Albala
BP: Blood pressure
DESERVE: Discharge Education Strategies for Reduction of Vascular Events
DBP: Diastolic blood pressure
FG: Franklin Glozah
IA: Isaac Ampomah
JB: Joel Birkemeier
KBTH: Korle Bu Teaching Hospital
LMICs: Low- and middle-income countries
NA: Noa Appleton
NCD: Non-communicable disease
NGOs: Non-governmental organizations
NHIS: National Health Insurance Scheme
NR: Nessa Ryan
PA: Philip Adongo
RA: Richard Adanu
SBP: Systolic blood pressure
TIA: Transient ischemic attack
TO: Temitope Ojo
